# PGE_1_-Containing Protocols Generate Mature (Leukemia-Derived) Dendritic Cells Directly from Leukemic Whole Blood

**DOI:** 10.3390/ijms20184590

**Published:** 2019-09-17

**Authors:** Daniel Christoph Amberger, Fatemeh Doraneh-Gard, Carina Gunsilius, Melanie Weinmann, Sabine Möbius, Christoph Kugler, Nicole Rogers, Corinna Böck, Uwe Ködel, Jan-Ole Werner, Doris Krämer, Britta Eiz-Vesper, Andreas Rank, Christoph Schmid, Helga Maria Schmetzer

**Affiliations:** 1 Medical Department 3, Working-group: Immune-Modulation, University Hospital Munich, 81377 Munich, Germany; daniel.amberger@gmx.at (D.C.A.); nikoo_792000@yahoo.com (F.D.-G.); Carina_Gunsilius@web.de (C.G.); melli.weinmann@hotmail.de (M.W.); sabine.moebius@tum.de (S.M.); christophkugler@gmx.net (C.K.); nicimunrog@yahoo.de (N.R.); corinna.boeck@uk-erlangen.de (C.B.); 2 Department of Neurology, Klinikum Großhadern, Ludwig-Maximilians-University, 81377 Munich, Germany; Uwe.Koedel@med.uni-muenchen.de; 3 Department of Hematology and Oncology, University Hospital of Tuebingen, 72076 Tuebingen, Germany; Jan-ole.werner@klinikum-nuernberg.de; 4 Department for Hematology and Oncology, University Hospital of Oldenburg, 26133 Oldenburg, Germany; onko.med@bhv.ameos.de; 5 Institute for Transfusion Medicine, Hannover Medical School, 30625 Hannover, Germany; Eiz-Vesper.Britta@mh-hannover.de; 6 Department of Hematology and Oncology, University Hospital of Augsburg, 86156 Augsburg, Germany; andreas.rank@klinikum-augsburg.de (A.R.); christoph.schmid@klinikum-augsburg.de (C.S.)

**Keywords:** PGE_1_, AML, leukemia-derived dendritic cells, immunotherapy, dendritic cells

## Abstract

Dendritic cells (DCs) and leukemia-derived DC (DC_leu_) are potent stimulators of various immunoreactive cells and they play a pivotal role in the (re-) activation of the immune system. As a potential treatment tool for patients with acute myeloid leukemia, we developed and analyzed two new PGE_1_-containing protocols (Pici-_PGE1_, Kit M) to generate DC/DC_leu_ ex vivo from leukemic peripheral blood mononuclear cells (PBMCs) or directly from leukemic whole blood (WB) to simulate physiological conditions. Pici-_PGE1_ generated significantly higher amounts of DCs from leukemic and healthy PBMCs when compared to control and comparable amounts as the already established protocol Pici-_PGE2_. The proportions of sufficient DC-generation were even higher after DC/DC_leu_-generation with Pici-_PGE1_. With Kits, it was possible to generate DCs and DC_leu_ directly from leukemic and healthy WB without induction of blast proliferation. The average amounts of generated DCs and DC_leu_-subgroups were comparable with all Kits. The PGE_1_ containing Kit M generated significantly higher amounts of mature DCs when compared to the PGE_2_-containing Kit K and increased the anti-leukemic-activity. In summary PGE_1_-containing protocols were suitable for generating DC/DC_leu_ from PBMCs as well as from WB, which reliably (re-) activated immunoreactive cells, improved the overall ex vivo anti-leukemic activity, and influenced cytokine-release-profiles.

## 1. Introduction

Acute myeloid leukemia (AML) is a clonal disease that is characterized by an uncontrolled proliferation and an impaired differentiation of myeloid progenitor cells (blasts) with overall five-year-survival rates of about 28.3 % [1,2]. In the last few decades, the therapy with antigen presenting cells (APCs), such as dendritic cells (DCs) revolutionized immunotherapy of AML [3,4,5].

APCs play a pivotal role in connecting the innate and the adaptive immune system with the properties to migrate into different tissues and activate different immune reactive cells. They internalize and process antigens, present antigen-fragments via major histocompatibility complex (MHC), and form immunological synapses with T cells, resulting in a clonally restricted and potent T cell-activation [6,7,8,9,10]. 

Two different DC-based immunotherapy strategies have been developed. Monocyte (CD14^+^) derived DCs can be generated with different response modifiers in cultures and they are loaded with leukemic-associated-antigens (LAA) by the electroporation of messenger ribonucleic acid (mRNA) or by peptide pulsing [11,12,13]. After expensive ex vivo manipulation and the production of cells under Good Manufacturing Practice (GMP), DCs can be (re-) administrated to patients as a vaccine [14,15].

Moreover, leukemic blasts can be converted ex vivo directly to leukemia derived DC (DC_leu_), presenting the whole leukemic antigen repertoire. DC_leu_ simultaneously express DC-antigens and -individual patients’ blast markers (blast-antigens) [16]. 

DCs and DC_leu_ can be generated ex vivo from leukemic peripheral blood mononuclear cells (PBMCs) or whole blood (WB) without the induction of blast proliferation [17,18,19]. DC/DC_leu_-generating-protocols contain combinations of different response modifiers, including (1) cytokines such as granulocyte-macrophage colony-stimulating factor (GM-CSF) or Interleukin 4 (IL-4) that induce differentiation of myeloid progenitor cells; or (2) Calcium-Inophore (A23187) as a cytokine free DC/DC_leu_-generating method; (3) bacterial or nucleic stimulation with danger signaling effects, such as Picibanil (OK432), a lysis product from the streptococcus pyogenes or Polyinosinic:polycytidylic acid (poly I:C); and, (4) substances inducing maturation of DC/DC_leu_, such as Prostaglandin E_2_ (PGE_2_) or Tumor-necrosis-factor alpha (TNF-α) [17,20]. Furthermore, Interleukin 1 beta (IL-1β), Interleukin 6 (IL-6), Interferon gamma (IFN-γ), Interferon alpha (IFN-α), and Fms-related tyrosine kinase 3 ligand (FLT3-L) were used and analyzed in different protocols to generate DCs and/or DC_leu_ from leukemic or healthy PBMCs [17,20,21,22,23,24]. 

The stimulation of T cell enriched immunoreactive cells with DC/DC_leu_ ex vivo in a mixed lymphocyte culture (MLC) regularly results in the (re-) activation of T cells against leukemic blasts, although depending on the DC/DC_leu_ protocol used for the generation of DC/DC_leu_ [17,25,26]. We could already show that proliferating T cells (T_prol_ CD71^+^, CD69^+^), non-naïve T cells (T_non-naïve_, CD45RO^+^), regulatory T cells (T_reg_, CD25^++^CD127^low^), β-integrin^+^ T cells and T cells with effector function, such as central-memory T cells (T_cm_, CD45RO^+^CCR7^+^), effector (memory) T cells (T_eff-em_, CD45RO^+^CCR7^−^) increase, while naïve T cells (T_naive_, CD45RO^−^) decrease during MLC [27,28]. DC/DC_leu_ probably contribute to stimulating and activating cells from the innate immune system and cells on the interface of the innate and the adaptive immune system, such as natural killer cells, invariant natural killer cells, or cytokine induced killer cells [29]. 

Soluble factors (e.g., cytokines) are involved in the leukemogenesis, as well as in anti-leukemic immunoreactions, thereby influencing the persistence or elimination of AML-cells and patients’ treatment outcome [30]. Monocyte chemotactic protein 1 (MCP-1, also known as CC-chemokine ligand 2, CCL-2) is an inflammatory cytokine, with antitumor activity [31]. Interleukin 17A (IL17-A) is classified as an anti-tumor response related cytokine and Interleukin 10 (IL-10) is characterized as an anti-inflammatory cytokine [32,33,34,35].

Prostaglandins are responsible and involved in different physiological functions, such as inflammation, regulation of renal-blood circulation, induction of fever, and the protection of the gastric mucosa from gastric acid [36,37,38,39]. PGE_2_ and Prostaglandin E_1_ (PGE_1_) are arachidonic acid derivatives, which are synthesized via the cyclooxygenase 1 and 2 pathway (COX1 and COX2) [40]. The biochemical differences between PGE_1_ and PGE_2_ are caused by different amounts of double bounds in the side chain [41]. PGE_2_ (e.g., Dinoproston) is approved by the US Food and Drug Administration (FDA) for the induction of labor in cases with medical or obstetrical indication. Drugs that contain PGE_1_ are approved for the risk reduction of gastrointestinal ulcers during the treatment with nonsteroidal anti-inflammatory drugs (NSAIR) (e.g., Misoprostol) and to treat erectile dysfunction (e.g., Alprostadil). Furthermore, PGE_1_ is used to treat peripheral arterial disease and to maintain the patency of the ductus arteriosus in patients with ductal-dependent cardiac lesions [42,43,44]. PGE_1_ was also analyzed in a combination with heparin to prevent liver veno-occlusive disease (VOD), which is a life-threatening obliteration of hepatic venules, in patients with AML after bone marrow transplantation (BMT) [45,46]. 

To improve the DC/DC_leu_-treatment of patients with AML, we have developed minimalized Kits, containing combinations of at least two response modifiers. Following our hypotheses Kits should be able to convert leukemic blasts directly to DC/DC_leu_ in WB cultures. With respect to clinical applications this could mean, that patients could be directly treated with Kits, thereby inducing DC/DC_leu_-generation in vivo, which would render an adoptive transfer of ex vivo generated DC/DC_leu_ unnecessary. 

The aim of this study was

(1) to develop and to functionally evaluate a new PGE_1_-containing DC/DC_leu_ generating protocol to produce DCs and DC_leu_ from healthy and leukemic PBMCs;
(2) to develop and to functionally evaluate an immunomodulatory Kit M (containing GM-CSF and PGE_1_) to produce DCs and DC_leu_ directly from healthy and leukemic WB, thereby simulating in vivo conditions;
(3) to deduce an optimized protocol for the ex vivo generation of DC/DC_leu_ which might be used for an adoptive cell transfer; and,
(4) to deduce immunomodulatory Kits that might be able to convert myeloid leukemic blasts in vivo to DC/DC_leu_.

## 2. Results

### 2.1. Prolog

In the first part of this manuscript, we present a new PGE_1_-containing protocol (Pici-_PGE1_) for the generation of DC/DC_leu_ from healthy and leukemic PBMCs.

In the second part, we simulated physiological conditions and generated DC/DC_leu_ with the DC/DC_leu_-generating protocols Pici-_PGE1_ and Pici-_PGE2_ and immunomodulatory Kits directly from healthy and leukemic WB. The compositions of Picis and Kits are shown in Table 1 We correlated data with the DC/DC_leu_ stimulatory potential of T cell enriched immunoreactive cells and with the potential to generate anti-leukemia directed T cells as well as with cytokine-release-profiles.

### 2.2. DC/DC_leu_-Generation from Healthy and Leukemic PBMCs

#### 2.2.1. Significantly Higher Amounts of DCs Generated from Healthy and Leukemic PBMCs with Pici-_PGE1_ and Pici-_PGE2_ Compared to Controls

**Figure 1:**
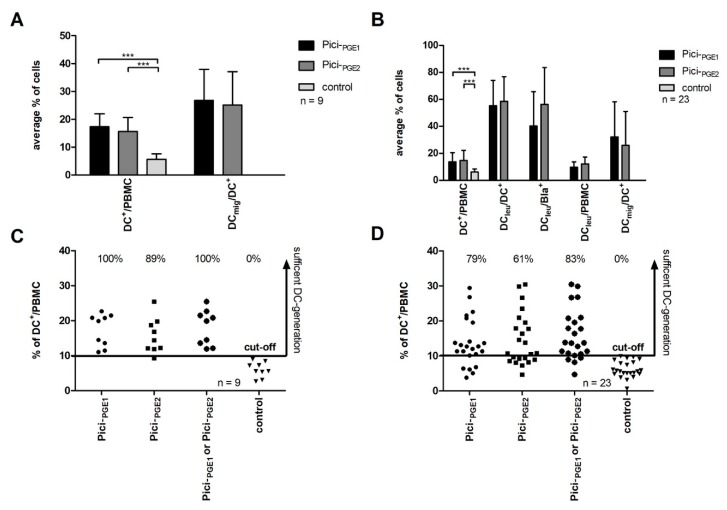
DC/DC_leu_-generation from healthy (left side) and leukemic peripheral blood mononuclear cells (PBMCs) (right side). (**A**) shows the average amounts ± standard deviation of generated dendritic cells (DCs) in the PBMC-fraction and mature DCs in the DC-fraction [CD197^+^DC^+^, (DC_mig_/DC^+^)] from healthy PBMCs with Pici-_PGE1_, Pici-_PGE2_ and control without added cytokines. (**B**) presents the average amounts ± standard deviation of generated DCs in the PBMC-fraction, DC_leu_-subgroups [including DC_leu_ in the DC-fraction (DC_leu_/DC^+^), DC_leu_ in the blast-fraction (to quantify amounts of leukemic blasts converted to DC_leu_) (DC_leu_/Bla^+^), DC_leu_ in the PBMC-fraction (DC_leu_/PBMC)] and DC_mig_ in the DC-fraction (DC_mig_/DC^+^) from leukemic PBMCs with Pici-_PGE1_, Pici-_PGE2_ and control without added cytokines. (**C**) and (**D**) show the percentages of sufficient DC-generation from healthy (**C**) and leukemic (**D**) PBMCs with Pici-_PGE1_, Pici-_PGE2_, Pici-_PGE1_ or Pici-_PGE2_ and control without added response modifiers according to cut-off-values (≥10% DC^+^/PBMC). Each dot (● ▪ ● ▼) represents DC-proportions generated from each individual healthy volunteer or AML-patient. DCs dendritic cells; DC_leu_ leukemic derived dendritic cells; PBMCs peripheral blood mononuclear cells. The differences were considered as significant*** with *p* values <0.005.

With Pici-_PGE1_ and Pici-_PGE2_ we generated on average significantly*** higher amounts of DCs from healthy PBMCs as compared to controls (*n* = 9) (Pici-_PGE1_: 17.4 ± 4.7% DC^+^/PBMC, *p* < 0.00003; Pici-_PGE2_: 15.6 ± 5.1% DC^+^/PBMC, *p* < 0.0003; control: 6.0 ± 2.2% DC^+^/PBMC). Although differences were not significant, we found, on average, higher amounts of DC^+^/PBMC after the stimulation of healthy PBMCs with Pici-_PGE1_ when compared to Pici-_PGE2_. No significant differences were found in amounts of DC_mig_/DC^+^ with Pici-_PGE1_ and Pici-_PGE2_ (26.8 vs. 25.1% DC_mig_/DC^+^, *p* < 0.77) (Figure 1A). 

We generated DCs and DC_leu_ from leukemic PBMCs and found, on average, significantly*** higher amounts of DC^+^/PBMC after culture with Pici-_PGE1_ and Pici-_PGE2_ compared to controls (*n* = 23) (Pici-_PGE1_: 13.7 ± 6.8% DC^+^/PBMC, *p* < 0.00003; Pici-_PGE2_: 14.7 ± 7.5% DC^+^/PBMC, *p* < 0.00002, control 6.1 ± 2.3% DC^+^/PBMC). No significant differences in the amounts of DC^+^/PBMC were found between Pici-_PGE1_ and Pici-_PGE2_ (*p* < 0.65). We found (not significantly) higher amounts of DC_mig_/DC^+^ after culture with Pici-_PGE1_ compared to Pici-_PGE2_ (32.1 vs. 25.9% DC_mig_/DC^+^, *p* < 0.35) (Figure 1B). Moreover, we could show that subtype (primary or secondary AML) and stage of the AML did not have an impact on the generation of DCs and DC_leu_ from leukemic PBMCs with Pici-_PGE1_ or Pici-_PGE2_ (data not shown). 

In summary, we conclude that DCs and DC_mig_ can be generated with Pici-_PGE1_ and Pici-_PGE2_ in comparable amounts from healthy and leukemic PBMCs. 

#### 2.2.2. Efficiency of Sufficient DC-Generation is Higher with Pici-_PGE1_ Compared to Pici-_PGE2_ from Leukemic PBMCs

In healthy and leukemic control groups, we found, in every given case, less than 10% DC^+^/PBMC. Therefore, we defined a cut-off value of ≥10% DC^+^/PBMC as a successful DC-generation from healthy and leukemic PBMCs. According to this cut-off value a successful DC-generation from healthy PBMCs was possible in 100% of cases (nine of nine cases) with Pici-_PGE1_ and in 89% of cases (eight of nine cases) with Pici-_PGE2_ (Figure 1C).

A sufficient DC-generation from leukemic PBMCs was possible in 79% of cases (18 of 23 cases) with Pici-_PGE1_ and in 61% of cases (14 of 23 cases) with Pici-_PGE2_. In 83% of cases, a sufficient DC-generation was possible with Pici-_PGE1_ or Pici-_PGE2_ (19 of 23 cases) (Figure 1D). In all cases with successful DC-generation, the amounts of DC_leu_ were comparable with Pici-_PGE1_ (*n* = 18) and Pici-_PGE2_ (*n* = 14). With Pici-_PGE1_ we generated on average 55.3 ± 18.8% DC_leu_/DC^+^ and Pici-_PGE2_ 58.5 ± 18.2% DC_leu_/DC^+^. The average amounts of blasts converted to DC_leu_ (DC_leu_/Bla^+^) were 40.3 ± 25.4% DC_leu_/Bla^+^ with Pici-_PGE1_ and 56.3 ± 27.2% DC_leu_/Bla^+^ with Pici-_PGE2_. 9.7 ± 4.0% DC_leu_/PBMC could be generated with Pici-_PGE1_ and 12.2 ± 5.1% DC_leu_/PBMC with Pici-_PGE2_ (Figure 1B).

In summary, the efficiencies of a sufficient DC-generation from leukemic PBMCs are higher with Pici-_PGE1_ as compared to Pici-_PGE2_ and comparable to healthy PBMCs. In four cases, no sufficient DC-generation was possible with both protocols. 

#### 2.2.3. Pici-_PGE1_ and Pici-_PGE2_ Do Not Induce Blast Proliferation During DC/DC_leu_-Culture from Leukemic PBMCs

After DC/DC_leu_-culture from leukemic PBMCs, we found on average comparable amounts of proliferating blasts that were not converted to DC_leu_ (Bla_prol-CD71_) with Pici-_PGE1_ or Pici-_PGE2_ as compared to control: Pici-_PGE1_: 26.8 ± 19.9%, *p* < 0.29; Pici-_PGE2_: 25.6 ± 16.8%, *p* < 0.39; control: 21.3 ± 14.7%. Comparable distributions were found for Bla_prol-Ipo-38_ (data not shown). 

We conclude that neither Pici-_PGE1_ nor Pici-_PGE2_ induce proliferation of blasts not converted to DC_leu_.

### 2.3. DC/DC_leu_-Generation from Healthy and Leukemic WB

#### 2.3.1. Comparable DC-Amounts can be Generated with Immunomodulatory Kits and Picis

We compared the DC^+^/WB values generated with Kit M, Kit K, and Kit I (Kits *n* = 27) and DC^+^/WB values that were generated with Pici-_PGE1_ and Pici-_PGE2_ from leukemic WB (Picis *n* = 18). 

**Figure 2:**
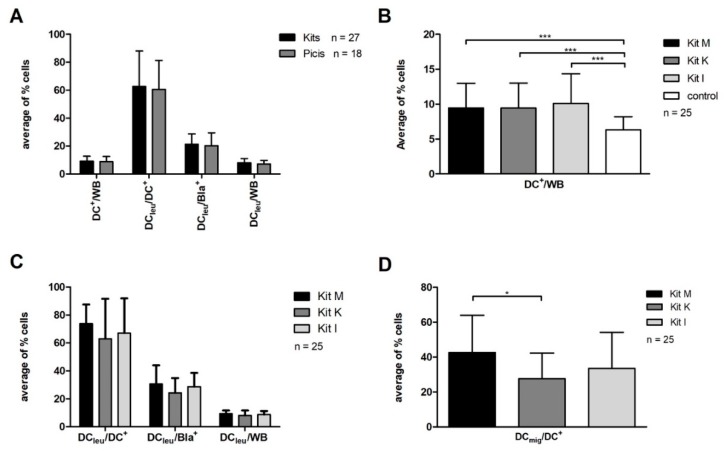
DC/DC_leu_-generation from leukemic whole blood (WB). (**A**) shows average amounts ± standard deviation of DC- and DC_leu_-proportions [including DC_leu_-subgroups: DC_leu_ in the DC-fraction (DC_leu_/DC^+^), DC_leu_ in the blast-fraction (to quantify amounts of leukemic blasts converted to DC_leu_) (DC_leu_/Bla^+^) and DC_leu_ in the WB-fraction (DC_leu_/WB)] from leukemic WB with Kits (including Kit M, Kit K, Kit I) compared to protocols Picis (including Pici-_PGE1_, Pici-_PGE2_). (**B**) shows average amounts ± standard deviation of generated DCs with Kit M, Kit K and Kit I compared to control. (**C**) presents average amounts ± standard deviation of generated DC_leu_ subgroups [including DC_leu_-subgroups DC_leu_ in the DC-fraction (DC_leu_/DC^+^), DC_leu_ in the blast-fraction (to quantify amounts of leukemic blasts converted to DC_leu_) (DC_leu_/Bla^+^) and DC_leu_ in the WB-fraction (DC_leu_/WB)] with Kit M, Kit K and Kit I. (**D**) shows average amounts ± standard deviation of DC_mig_/DC^+^ generated with Kit M, Kit K and Kit I. DC_mig_ are characterized by the expression of CCR7. DCs dendritic cells; DC_leu_ leukemic derived dendritic cells; WB whole blood. The differences were considered as significant*, with *p* values between 0.1 and 0.05 and as significant*** with *p* values <0.005.

Amounts of generated DC^+^/WB from leukemic WB-samples were not significantly different in both groups (Kits: 9.3 ± 3.4% DC^+^/WB vs. Picis: 8.8 ± 3.7% DC^+^/WB, *p* < 0.62). Comparable results were found after the DC-generation from healthy WB (data not shown). Moreover, we found comparable amounts of DC_leu_/DC^+^, DC_leu_/Bla^+^, and DC_leu_/WB with Kits as compared to Picis (DC_leu_/DC^+^: 62.7 ± 25.30% vs. 60.6 ± 20.8%; DC_leu_/Bla^+^: 21.3 ± 7.4% vs. 20.3 ± 9.1%; DC_leu_/WB: 8.1 ± 3.0% vs. 7.2 ± 2.6%) (Figure 2A). 

We summarize that DC/DC_leu_-generation (including subgroups) is possible in comparable amounts with Kits from leukemic and healthy WB when compared to Picis. 

#### 2.3.2. Significantly Higher Amounts of DC^+^/WB Generated from Healthy and Leukemic WB with Immunomodulatory Kit M, Kit K and Kit I Compared to Control

We generated DCs from leukemic WB and found, on average, significantly*** higher amounts of DC^+^/WB after WB-DC/DC_leu_-cultures with Kit M, Kit K, and Kit I as compared to control (*n* = 25) (Kit M: 9.5 ± 3.6% DC^+^/WB, *p* < 0.0004; Kit K: 9.5 ± 3.6% DC^+^/WB, *p* < 0.0004; Kit I: 10.1 ± 4.4% DC^+^/WB, *p* < 0.0003; control: 6.3 ± 1.9% DC^+^/WB) (Figure 2B). Comparable distributions were found after DC-cultures from healthy WB (*n* = 9, data not shown). In the comparison of Kit M, Kit K, and Kit I we found, on average, comparable amounts of generated DCs in the WB-fraction from healthy and leukemic WB. In DC_leu_-subgroups, including DC_leu_/Bla^+^, DC_leu_/DC^+^, as well as DC_leu_/WB no significant differences were found (Figure 2C). 

With Kit M, significantly* higher amounts of DC_mig_/DC^+^ could be generated when compared to Kit K (*n* = 25): (42.6 ± 21.5% DC_mig_/DC^+^ vs. 27.6 ± 14.7% DC_mig_/DC^+^; *p* < 0.09). In the comparison of Kit M and Kit I, no significant differences were found (Kit I: 33.5 ± 20.7% DC_mig_/DC^+^; *p* < 0.35). (Figure 2D).

In summary, significantly higher amounts of DC^+^/WB can be generated with immunomodulatory Kits from healthy and leukemic WB as compared to control. The amounts of generated DC and DC_leu_-subgroups are comparable with all three Kits, with the exception of significantly higher frequencies of DC_mig_/DC^+^ after DC/DC_leu_-culture with Kit M as compared to Kit K. 

#### 2.3.3. Kits Do Not Induce Blast-Proliferation Compared to Control

We found comparable amounts of proliferating blasts that were not converted to DC_leu_ (Bla_prol-CD71_) in the WB-fraction after culture of leukemic WB with Kit M, Kit K, Kit I, and control (*n* = 24): Kit M: 8.5 ± 13.0%, *p* < 0.48; Kit K: 8.4 ± 11.0%, *p* < 0.45; Kit I: 9.9 ± 10.7%, *p* < 0.18 and control: 6.5 ± 6.1%. Comparable distributions were found for Bla_prol-Ipo-38_ (data not shown). 

In summary, Kits do not induce blast proliferation during DC/DC_leu_-culture from leukemic WB when compared to control. 

### 2.4. Stimulatory Effect of DC/DC_leu_ (Generated with Kits from Leukemic WB) on T Cell Enriched Immunoreactive Cells in MLC and the Corresponding Blast-Lysis Activity

#### 2.4.1. Comparison of T Cell Amounts, Phenotypes, and Blast Lysis Activity in Uncultured Cells, after MLC^WB-DC Kits^ and after MLC^WB^

As shown above, DC- and DC_leu_-generation was possible from healthy and leukemic WB with Kits. Here we studied the (potential) stimulating effect of generated DCs and DC_leu_ on T cell enriched immunoreactive cells in MLC. Therefore, we compared T cell compositions in uncultured cells (*n* = 11) (uncultured cells) with those after stimulation in MLC^WB-DC-Kit M^, MLC^WB-DC-Kit K^, and MLC^WB-DC-Kit I^ (*n* = 24) (MLC^WB-DC Kits^) and after stimulation in MLC^WB^ (*n* = 11) (MLC^WB^) as control. 

**Figure 3:**
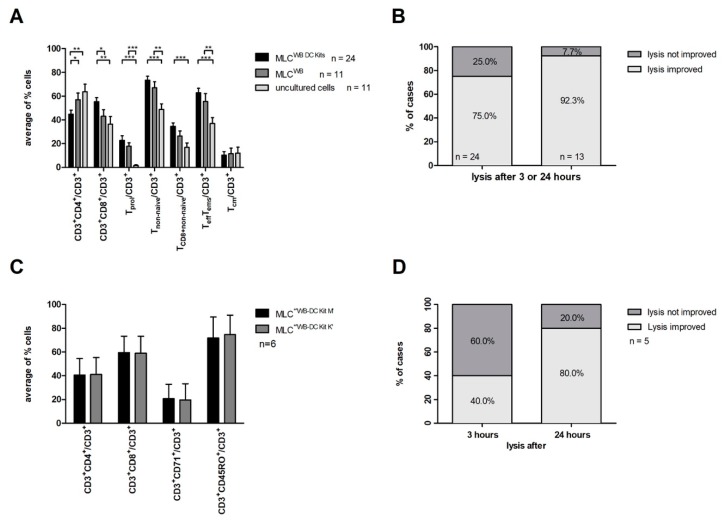
Stimulatory effect of DC/DC_leu_ generated with Kits from leukemic WB on T cell enriched immunoreactive cells in MLC and the corresponding blast-lysis activity. (**A**) shows average amounts ± standard deviation of T cell subsets after stimulation of T cell enriched immunoreactive cells with DC/DC_leu_ generated with Kits from leukemic WB (MLC^WB-DC Kits^), after stimulation of T cell enriched immunoreactive cells with a WB cell suspension not pretreated with Kits (MLC^WB^) and in uncultured cells. Cells were analyzed by flow cytometry and referred to the CD3^+^ cell fraction. (**B**) shows the improvement of blast-lysis-activity compared to controls after 3 or 24 h of co-culture of target and effector cells (left column) and of cases with improved blast lysis with at least one MLC^WB-DC Kit^ proportions compared to MLC^WB^ in each individual patient (right column). (**C**) shows average amounts ± standard deviation of different T cell subsets after MLC^WB-DC Kit M^ and MLC^WB-DC Kit K^. (**D**) shows percentage of cases with improved blast-lysis after MLC^WB-DC Kit M^ compared to MLC^WB-DC Kit K^ after 3 h (left column) and after 24 h (right column). MLC mixed lymphocyte culture; WB whole blood. The differences were considered as significant*, with *p* values between 0.1 and 0.05, as significant** with *p* values between 0.05 and 0.005 and as significant*** with *p* values <0.005.

In general, we found a significantly higher activation status of immunoreactive T cells after MLC^WB-DC Kits^ as compared to MLC^WB^ and uncultured T cells. The main findings were characterized by increased amounts of CD3^+^CD8^+^/CD3^+^ and the corresponding decrease of CD3^+^CD4^+^/CD3^+^ after MLC^WB-DC Kits^ when compared to MLC^WB^. We found after MLC^WB-DC Kits^ as well as after MLC^WB^ increased amounts of proliferating T cells when compared to uncultured cells, and also a shift from naive to non-naïve T cell subsets. Detailed results are shown in Table 2A and in Figure 3A. Since IL-2 was added to all MLC-experiments (including MLC^WB^), amounts of T cell subsets also increased after MLC^WB^. 

Furthermore, we pooled all the results obtained with the cytotoxicity assay after MLC^WB-DC Kit^ (including MLC^WB-DC Kit M^, MLC^WB-DC Kit K^, MLC^WB-DC Kit I^) and compared the results to MLC^WB^ after 3 or 24 h (*n* = 24) of incubation of effector with target cells. Anti-leukemic activity was defined as lysis of blast-target-cells obtained either after 3 or 24 h. Moreover, we analyzed the anti-leukemic activity in each individual patient (*n* = 13).

After MLC^WB-DC^ lysis of blasts target cells could be improved in 75.0% of cases (18 of 24 cases) as compared to MLC^WB^ after 3 or 24 h. 

Furthermore, in 92.3% of patients (12 of 13 patients) we could select at least one MLC^WB-DC Kit^ in each individual patient, which improved the blasts lysis after 3 h or 24 h when compared to MLC^WB^ (Figure 3B).

In summary, we regularly demonstrate an increase of T cells’ anti-leukemic activity after stimulation with DC/DC_leu_ generated with Kits from leukemic WB. 

#### 2.4.2. Comparison of T Cell Amounts, Phenotypes and Blast Lytic Activity after MLC^WB-DC Kit M^ and MLC^WB-DC Kit K^

DC/DC_leu_-generation was possible with Kits from leukemic WB and the stimulation of T cell enriched immunereactive cells with these DC/DC_leu_ resulted in T cell activation. The premise for this analysis was that MLC^WB-DC Kit M^ and MLC^WB-DC Kit K^ (*n* = 6) were performed in parallel in patients with the corresponding leukemic WB-samples. 

As shown in Figure 3C and Table 2B, we found, on average, comparable amounts of T cells (subsets), including CD3^+^CD8^+^/CD3^+^, CD3^+^CD4^+^/CD3^+^ cells and CD3^+^CD71^+^/CD3^+^ cells after MLC^WB-DC Kit M^ as compared to ML^WB-DC Kit K^ from leukemic WB. Furthermore, no significant differences were found for the non-naïve or naïve T cells as well as CD3^+^CD45RO^+^CCR7^−^/CD3^+^ and CD3^+^CD45RO^+^CCR7^+^/CD3^+^ after MLC^WB-DC Kit M^ and MLC^WB-DC Kit K^ (data not shown). Comparable distributions were found after MLC^WB-DC Kit I^.

After 3 h of incubation of DC/DC_leu_ stimulated effector cells with blast target cells we found an improved lysis after MLC^WB-DC Kit M^ in 40% of cases (two of five cases) when compared to MLC^WB-DC Kit K^. Blast lysis was improved in 80% of cases (four of five cases) after 24 h after MLC^WB-DC Kit M^ compared to MLC^WB-DC Kit^ (Figure 3D). 

We conclude that DC/DC_leu_ in a MLC^WB–DC^ have an impact on the amounts of different T cell subsets after stimulation as compared to MLC^WB^ and uncultured cells, however T cell compositions after MLC^WB-DC Kits^ were comparable with Kits used to generate DC/DC_leu._ Furthermore, we conclude that blast-lysis was improved in all cases (except one) after MLC^WB-DC Kit M^ when compared to MLC^WB-DC Kit K^ after 24 h.

### 2.5. Cytokine-Release-Profiles in Serum and after WB DC/DC_leu_-Culture

Cytokine releases by blasts, DCs, as well as by immunoreactive cells are known to influence immunological as well as immune-escape-reactions. Therefore, we correlated cytokine releases in DC/DC_leu_-culture-supernatants as well as in serum. 

#### 2.5.1. Significantly Higher Concentrations of the Inflammatory Cytokine and Antitumor Response Related Cytokine Found after WB-DC/DC_leu_-Culture with Kits Compared to Serum

We studied the cytokine release levels after WB-DC/DC_leu_-culture with Kits and in serum. Therefore, we pooled all of the results after WB-DC/DC_leu_-culture with Kits (Kit M, Kit K and Kit I) (*n* = 27) from leukemic WB and compared it to cytokine concentrations in serum (*n* = 8). 

**Figure 4:**
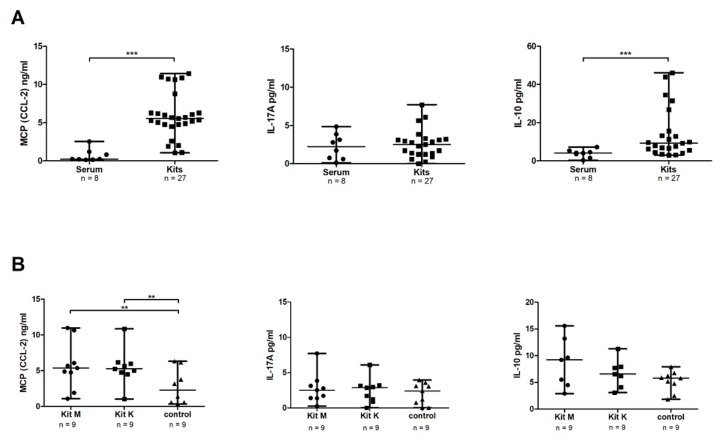
Cytokine-release-profiles after DC/DC_leu_-culture from leukemic WB. (**A**) shows concentrations of MCP-1 (CCL-2) (ng/mL), IL-17A (pg/mL) and IL-10 (pg/mL) in serum and after DC/DC_leu_-culture from leukemic WB with Kits (including Kit M, Kit K, and Kit I). (**B**) shows the comparison of concentrations of MCP-1 (CCL-2), IL-17A, and IL-10 after DC/DC_leu_-culture from leukemic WB with Kit M, Kit K and control without added cytokines. All cytokines were measured with ELISA. MCP-1 Monocyte chemotactic protein 1; CCL-2 CC-chemokine ligand 2; IL17-A Interleukin 17A; IL-10 Interleukin 10. The differences were considered as significant** with *p* values between 0.05 and 0.005 and as significant*** with *p* values <0.005.

We found significantly*** higher median concentrations of MCP-1 (CCL-2) after WB-DC/DC_leu_-culture with Kits as compared to serum [5.5 ng/mL (range: 1.0–11.4) vs. 0.2 ng/mL (range: 0.1–2.5), *p* < 0.000001]. Furthermore, we found significantly*** higher median concentrations of the anti-inflammatory cytokine IL-10 after DC/DC_leu_-culture [9.2 pg/mL (range: 2.9–46.1) vs. 4.1 pg/mL (range: 0.3–4.1), *p* < 0.01]. No significant differences were found for the cytokine IL-17A (*p* < 0.6). Comparable results were found after DC-generation from healthy WB (data not shown) (Figure 4A). 

Furthermore, we compared the cytokine concentrations in supernatants after DC/DC_leu_-cultures from leukemic WB with Kits and with Picis. In both groups, comparable concentrations of MCP-1 (CCL-2), IL-17A, and IL-10 were found (data not shown). 

#### 2.5.2. Significantly Higher Concentrations of MCP-1 (CCL-2) Found after DC/DC_leu_-Culture with Kit M and Kit K Compared to Control

We compared cytokine secretion after DC/DC_leu_-culture of leukemic WB with Kits when compared to control without added response modifiers. We found significantly** higher median concentrations of MCP-1 (CCL-2) after DC/DC_leu_-culture with Kit M and Kit K as compared to control (*n* = 9) [Kit M: 5.4 ng/mL (range: 1.1–11.0), *p* < 0.06; Kit K: 5.3 ng/mL (range: 1.0–10.9), *p* < 0.04; control: 2.3 ng/mL (range: 0.4–6.3)]. For the cytokines IL-17A and IL-10, no significant differences were found as compared to controls (Figure 4B). Comparable results were found for the Kit I (data not shown).

We conclude that the addition of Kits to leukemic WB influences the cytokine release profiles when compared to control. Kit M and Kit K produce comparable cytokine-release-profiles.

## 3. Discussion

### 3.1. DC and DC_leu_ Based Immunotherapy for Patients with AML

DCs play a crucial role in the (re-) activation of the immune system and in linking the innate and adaptive immune system. These professional APCs have the ability to migrate into different tissues and to induce an immunological memory. During the last decades, different strategies have been developed to utilize DCs as a treatment tool for patients with AML. DCs can be generated ex vivo from CD14^+^ monocytes, loaded with different LAAs or tumor antigens, and they can be re-administrated to the patient as vaccine. It was already reported that vaccination with ex vivo generated monocyte-derived DCs increased the amounts of leukemia specific T cells in AML-patients and stabilized complete remission [15,48,49]. Unfortunately, the production of monocyte-derived DCs ex vivo is time-consuming, expensive, has to be performed under GMP-conditions, and the cell production is limited by the selection of LAA [11,14,15,50]. 

On the other hand, we and others could already show that clonal leukemic blasts can be regularly converted to DC_leu_ with different DC/DC_leu_-generating protocols, independent from age, FAB-classification, mutation or hematopoietic stem cell transplantation (HSCT) status, mutations of the disease, and FAB classification [17,51]. The clonal leukemic origin of DC_leu_ was already confirmed with fluorescence in situ hybridization (FISH) analysis [52]. 

### 3.2. The PGE_1_-Containing Protocol Pici-_PGE1_ is More Reliable to Generate DCs in Sufficient Amounts from Healthy and Leukemic PBMCs Compared to the PGE_2_-Containing Protocol Pici-_PGE2_

It was already shown that PGE_2_ produced by the enzyme cyclooxygenase 2 (COX-2) has a crucial role in tumor genesis in colorectal cancers in vivo [53,54]. It was reported that PGE_2_ influences the apoptosis, induces the angiogenesis, and activates the proliferation of cancer cells [55,56,57]. Whereas, in highly metastatic melanoma cells, it was shown that PGE_1_ promotes anti-tumor effects, such as the inhibition of invasion and cell growth [58]. Moreover, PGE_1_ increased the differentiation of tumor cells and decreased the expression levels of metalloproteinase (MMP) 2 and 9 [59]. Furthermore, it was shown that PGE_1_ increased the concentrations of Cis-Platin in cancers cells after treating rats with peritoneal carcinomatosis [60]. Transferring these findings into the AML treatment, we hypothesize that PGE_1_ could influence anti-cancer reactions in a positive way. Therefore, we analyzed the potential of PGE_1_-containing DC/DC_leu_-generating protocols to produce DCs and DC_leu_ from healthy and leukemic PBMCs as well as from WB. In the past few years it was shown, that it is possible to generate mature DCs and DC_leu_ from leukemic and healthy PBMCs with the PGE_2_-containing protocol Pici-_PGE2_ [17,20]. We could confirm this and we add, in addition, that the new protocol Pici-_PGE1_ produced comparable amounts of DCs and DC_leu_ with a higher efficiency of sufficient DC generation from healthy and leukemic PBMCs when compared to Pici-_PGE2_. These results suggest, that PGE_1_ mediates the maturation of DCs and DC_leu_ with comparable efficiency as PGE_2_, but might be also involved in the differentiation of myeloid progenitor cells and the production of DC/DC_leu_. This effect might be explained by the different mode of action of PGE_1_ and PGE_2_ on prostaglandin receptors (EP receptors) with different down streaming effects due to their different biochemical reactivities [61]. The generation of DC/DC_leu_ was possible with both protocols, independent of patients’ FAB-classification, stage (first diagnosis vs. relapse), or status (primary vs. secondary AML) of the disease. Moreover, we could confirm that neither Pici-_PGE1_ nor Pici-_PGE2_ induced the proliferation of non-converted blasts during DC/DC_leu_-cultures [19]. In four AML-cases, no sufficient DC/DC_leu_-generation was possible from leukemic PBMCs with Pici-_PGE2_ and Pici-_PGE1_, which might be due to various expressions of cytokine-receptors (e.g., GM-CSF receptors, stem cell-receptors) or prostaglandin-receptors (EP-receptors) on leukemic blasts [62,63]. These patients did not receive chemotherapy before conducting DC/DC_leu_ culture experiments, however spontaneous apoptosis or cell death of blood cells has to be discussed. Technical limitations were excluded. 

### 3.3. Successful Generation of DCs and DC_leu_ from Healthy and Leukemic WB Cultures

We established a WB-model to generate DC/DC_leu_ from leukemic WB containing all soluble and cellular factors of the individual patient to simulate physiological conditions. According to our hypothesis, DCs and DC_leu_ could also be generated in vivo by modulating myeloid blasts in their natural microenvironment after administrating a combination of different response modifiers (e.g., Kit M, Kit K, Kit I) to the patient. Therefore, well known DC/DC_leu_ generating protocols (e.g., Pici-_PGE1_, Pici-_PGE2_) were applied in WB settings and results that were obtained with Kit M, Kit K, or/and Kit I compared: our results show that DCs and DC_leu_ (including subgroups) could be generated in comparable amounts with Kits and Picis directly from WB [17,20]. Additionally, DC_leu_ subgroups did not significantly differ. We conclude that DCs and DC_leu_ can be generated with Kits from leukemic WB ex vivo. The fact that significantly more DC/DC_leu_ could be generated with Kit M, Kit K, and Kit I when compared to control without added response modifiers points to the fact that the combination of two response modifiers is necessary to generate DCs and DC_leu_ in sufficient amounts directly from leukemic WB: the induction of hematopoietic differentiation is induced by GM-CSF, danger signaling, and/or maturation signaling of DC/DC_leu_ is caused by PGE_1_, PGE_2_, or Picibanil. When compared to PBMC—cultures, response modifiers, such as IL-4 and other/unknown cytokines, are physiological components of the microenvironment in WB, and therefore they have not to be added or included in DC/DC_leu_-generating protocols [35]. The average amounts of generated DCs (including DC_leu_ subgroups) were comparable with all three Kits, although amounts of matured DCs were significantly higher after WB-treatment with the PGE_1_-containing Kit M as compared to the PGE_2_-containing Kit K. This finding might also be explained by the different mode of action of PGE_1_ and PGE_2_ on EP receptors with different down streaming effects [61]. Especially, for the in vivo use of DC/DC_leu_, the expression of the lymph-node-homing-receptor CCR7 (marker for mature DCs) is crucial for the migratory capacity of DCs and DC_leu_ to the lymph node, where they activate T cells and other immunoreactive cells and induce anti-tumor/anti-leukemic activity [64,65,66]. Comparable results were already found under hypoxic conditions [67]. The proliferation of blasts (not converted to DC_leu_) was not induced with Kits during DC/DC_leu_-cultures. Therefore, we conclude that Kits might be safe tools for an in vivo treatment.

We could already show that, after the stimulation of T cell, enriched immunoreactive cells with DCs and DC_leu_ generated from leukemic PBMCs composition of T cells could be shifted and influenced in a positive way and that anti-leukemic activity could be induced [17,25,27,28]. T cells directly kill cancer-cells via the Granzyme B and Perforin pathway and/or indirectly through the secretion of IFN-γ or tumor-necrosis-factor-alpha (TNF-α). We found after MLC^WB DC-Kits^ a higher activation status with increased amounts of proliferating T cells, as well as a shift from naive to non-naïve T cell subsets when compared to cells before culture. When compared to MLC^WB^, amounts of CD3^+^CD8^+^ significantly increased. After MLC^WB^ higher amounts of different T cell subsets could also be found and they can be explained by IL-2 added to all MLC, which is a potent endogenous T cell and NK cell activating cytokine [68]. The induction of an immunological memory can be postulated due to the DC concept. We found comparable amounts of central memory T cells after MLC^WB-Kits^, MLC^WB^ and in uncultured cells. However, we found significantly more effector memory T cells after MLC^WB-Kits^, pointing to an induction of these specialized memory cells, which can enter different tissues to initiate inflammation and cytotoxicity [69]. We speculate that, in our setting, the incubation time to produce memory T cells after DC/DC_leu_-stimulation is too short, therefore only effector memory T cells are produced. The induction of memory T cells is crucial for the induction of long term remission in patients with AML. 

The composition of Kits to produce DC/DC_leu_, used for the stimulation of T cell enriched immunoreactive cells has no impact on the average amounts and phenotypes of T cell subsets after MLC, but on the anti-leukemic activity. The most important result of our study was that we could demonstrate that anti-leukemic activity could be improved after MLC^WB-DC-Kits^ compared to MLC^WB^. Increased production of leukemia-specific cells after MLC^WB-DC-Kits^ as compared to MLC^WB^ was shown in the proof of concept-assays. Extended leukemia-specific evaluations are part of our ongoing research. These results suggest that DC/DC_leu_ generated with Kits from leukemic WB induce the immune system and can activate specific anti-leukemic activity against leukemic blasts after MLC. Furthermore, we could show that the anti-leukemic activity is comparable and it might be superior after MLC DC/DC_leu_ generated with the PGE_1_-containing Kit M when compared to the PGE_2_-containing Kit K, especially after 24 h of simultaneous incubation of the effector and target cells. This finding might be explained by the fact that PGE_2_ could induce the expression of indoleamin 2,3-dioxgenase-1 (IDO1), an immunoregulatory enzyme that activates immunosuppressive T_reg_, but does not impair the antigen presentation capacity of DCs [70,71,72,73,74]. 

Moreover, during WB DC/DC_leu_-cultures with Kits as well as Picis cytokine-release-profiles were influenced compared to serum as well as compared to control. We can conclude that Kits induce the production of anti-tumor response related cytokines as well as inflammatory cytokines during DC/DC_leu_ cultures, and therefore improve anti-leukemic reactivity of T cells after stimulation with Kit treated WB. 

## 4. Material and Methods

### 4.1. Sample Collection

After obtaining written informed consent, in accordance with the Helsinki protocol and the local Ethic Committee (Pettenkoferstraße 8a, 80336 Munich, Germany, Ludwigs-Maximilians-University-Hospital in Munich; VoteNo 33905), the heparinized peripheral WB samples were taken from patients in acute phases of AML and from healthy volunteers. The University-Hospitals of Oldenburg, Tuebingen, Munich and Augsburg provided samples. Anticoagulation was performed with Lithium-heparin-tubes (7.5 mL, Sarstedt, Nuernberg, Germany) containing standardized concentrations of Heparin.

PBMCs were isolated from WB-samples by density gradient centrifugation while using the Ficoll-Hypaque-Technique (Biocoll-Separating-solution, Biochrom, Berlin, Germany) with a density gradient of 1.077 g/mL. PBMCs were washed and then suspended in phosphate-buffered saline (PBS, Biochrom, Berlin, Germany). CD3^+^T cells were enriched while using the MACS-technology (Milteney Biotech, Bergisch Gladbach, Germany). The purity of the viable T cells was, on average, 87.9% (range 77.4–96.1%). The viable cells were quantified while using Trypan Blue (Biochrom, Berlin, Germany) and they were counted with Neubauer-counting-chambers. PBMCs were directly used to set up DC/DC_leu_-cultures. T cells and the remaining PBMCs were used for subsequent experiments, therefore cells were frozen at −80 °C (using DMSO) and then thawed according to standardized protocols. Furthermore, the serum was frozen for subsequent ELISA-experiments. 

### 4.2. Patients’ Characteristics and Diagnostics

DC/DC_leu_ were generated from PBMC- and WB-samples that were obtained from AML patients (*n* = 29) and healthy volunteers (*n* = 10). The average age of AML patients was 58.6 years (range 21–79) and of healthy volunteers 28.1 years (range 20–56). The female to male ratio of AML patients was 1:1.2 and of healthy 1:0.4. The ages of healthy volunteers and AML patients were not age-matched, since no direct comparisons of those two groups were needed.

The diagnosis and classification of AML patients was based on the French-American-British (FAB) classification: AML without maturation (M1: *n* = 2), AML with granulocytic maturation (M2: *n* = 1), acute myelomonocytic leukemia (M4: *n* = 6), and acute monocytic leukemia (M5: *n* = 6). No FAB-classification was available in 14 AML cases. Patients presented with primary AML [pAML (*n* = 17)] or with secondary AML [sAML (*n* = 12)]. Patients’ stages were: first diagnosis (*n* = 20), relapse (*n* = 2) or relapse after HSCT (*n* = 4), and persisting disease (*n* = 3). Table 3 gives patients’ characteristics.

The cellular composition of AML- and healthy-peripheral-WB-samples as well as of AML- and healthy-PBMCs-samples are shown in Table 4. In cases with the aberrant expression of T-, B-, or monocytoid-antigens or CD56 on leukemic blasts, proportions of the corresponding cells were not included in the analysis. 

### 4.3. DC/DC_leu_-Generation from Isolated PBMCs

DC/DC_leu_ were generated from 3–4 × 10^6^ isolated healthy and leukemic PBMCs with the DC/DC_leu_-generating protocols Pici-_PGE1_ and Pici-_PGE2_ [17,20]. Therefore, cells were pipetted into 12-multiwell-tissue-culture-plates (ThermoFisher Scientific, Darmstadt, Germany) and they were diluted in 2 mL serum-free X-Vivo-15-medium (Lonza, Basel, Swiss). Cytokines were added, as described below. Half medium exchange was carried out after 3–4 cell culture days. A culture without added response modifiers served as a control.

### 4.4. DC/DC_leu_-Generation from WB

DC/DC_leu_ were generated from healthy and leukemic WB (presenting the physiological cellular and soluble composition of the individual samples) with the DC/DC_leu_-generating protocols Pici-_PGE2_, Pici-_PGE1_, Kit M, Kit K and Kit I [47]. Therefore, 500 μL WB were pipetted in 12-multiwell-plates and then diluted 1:2 in X-Vivo-15-medium (Lonza, Basel, Swiss) to imitate the physiological conditions. Response modifiers and immune-modulating factors were added to cultures, as described below. A culture without added response modifiers served as a control. All of the response modifiers used for the DC/DC_leu_-generation are approved for human treatment. Table 1 provides the compositions of DC/DC_leu_-generating protocols. 

All of the cell-culture-experiments were conducted at standard laboratory conditions comprising 37°C, 21% O_2_ and 5% CO_2_. At the day of harvest, cell culture supernatants were collected from DC/DC_leu_-cultures and MLC-cultures and frozen, according to the standardized protocols for subsequent ELISA-experiments.

#### 4.4.1. Picibanil-_PGE1_ (Pici-_PGE1_)

DC/DC_leu_ were generated from PBMCs and WB with the DC/DC_leu_-generating protocol Pici-_PGE1_ -containing 500 U/mL granulocyte-macrophage colony-stimulation factor (GM-CSF, Sanofi-Aventis, Frankfurt, Germany) and 250 U/mL Interleukin-4 (IL-4) (PeproTech, Berlin, Germany). After 6–7 days, 10μg/mL Picibanil (OK 432), a lysis product from Streptococcus pyogenes that has unspecific immune modulatory effects (Chugai Pharmaceutical Co., Kajiwara, Japan) and 1μg/mL Prostaglandin E_1_ (PGE_1_) (PeproTech, Berlin, Germany) were added. After 7–10 days of incubation, the cells were harvested and used for subsequent experiments.

#### 4.4.2. Picibanil-_PGE2_ (Pici-_PGE2_)

DC/DC_leu_ were generated from PBMCs and WB with the Pici-_PGE2_ DC/DC_leu_-generating protocol, with the same composition, as given above, for Pici-_PGE1_, however substituting PGE_1_ by PGE_2_ (PeproTech, Berlin, Germany) [17,20].

#### 4.4.3. Kit M:

The generation of DC/DC_leu_ from WB with Kit M was performed while using 800 U/mL GM-CSF and 1μg/mL PGE_1_ [47]. After 2–3 days the same amounts of cytokines were added and after in total 7–10 days of incubation cells were harvested and used for subsequent experiments.

#### 4.4.4. Kit K:

Kit K consisted of 800 U/mL GM-CSF and 1μg/mL PGE_2_ and it was used to generate DC/DC_leu_ from WB [47]. Incubations were performed in analogy to Kit M.

#### 4.4.5. Kit I:

DC/DC_leu_ were generated with Kit I from WB using 800 U/mL GM-CSF and 10 μg/mL Picibanil [47]. The incubations were performed in analogy to Kit M.

### 4.5. Cell-Characterization by Flow Cytometry

Flow cytometric analyses were carried out to evaluate and quantify the amounts and phenotypes of leukemic blasts, T cell subsets, B cells, monocytes and DC/DC_leu_ subsets in the PBMC- and WB-fractions before and after different cultures. Panels with several monoclonal antibodies (moAbs) labeled with Fluorescein isothiocyanat (FITC), phycoerythrin (PE), tandem Cy7-PE conjugation (Cy7-PE), or allophycocyanin (APC) were used. The antibodies were provided by Beckman Coulter, Krefeld, Germany (^a^), Becton Dickinson, Heidelberg, Germany (^b^), Miltenyi Biotech, Bergisch Gladbach, Germany (^c^), Thermo Fisher, Darmstadt, Germany (^d^) and Santa Cruz Biotechnology, Heidelberg, Germany (^e^). FITC-conjugated moAbs against CD3^a^, CD15^a^, CD33^a^, CD34^a^, CD45RO^a^, CD65^a^, CD71^a^, CD83^a^, and IPO-38^e^ were used. To detect CD3^a^, CD4^a^, CD19^a^, CD33^a^, CD34^a^, CD56^a^, CD80^b^, CD83^a^, CD117^a^, and CD206^a^ PE-conjugated moAbs were used. MoAbs against CD3^a^, CD4^a^, CD14^b^, CD15^b^, CD19^a^, CD33^a^, CD34^a^, CD56^a^, CD65^c^, CD80^b^, CD117^a^, and CD197^b^ were labeled with Cy7-PE. APC-labeled moAbs against CD3^a^, CD4^b^, CD14^a^, CD15^b^, CD34^a^, CD45RO^d^, CD56^a^, CD65^c^, CD69^b^, CD83^b^, CD86^g^, CD117^a^, CD206^b^, and CD209^b^ were used. 7AAD^b^ was used to detect dead cells. To stain intracellular antigens (e.g., IPO-38) the FIX & PERM^®^ Cell Fixation and Cell Permeabilization Kit (ThermoFisher Scientific, Darmstadt, Germany) was used.

Erythrocytes in WB samples were lysed while using Lysing-Buffer (BD, Heidelberg, Germany), according to the manufacturer’s instructions. To stain cells with moAbs, they were resuspended in PBS (Biochrom, Berlin, Germany), containing 10% fetal calf-serum (FCS, Biochrome, Berlin, Germany) to avoid unspecific bindings and they were incubated for 15 min. in the dark at room temperature. Afterwards, the cells were washed, centrifuged, and resuspended in 100–200 μL PBS. At least 5000 events were evaluated with a fluorescence-activated cell sorting Flow-Cytometer (FACSCalibur^TM^) and Cell-Quest-data-acquisition and analysis software (Becton Dickson, Heidelberg, Germany). The isotype controls were conducted according to manufacturer’s instructions.

**Figure 5:**
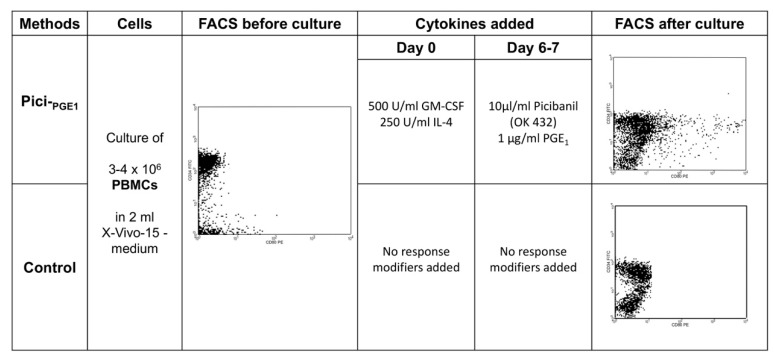
Workflow and FACS analysis of DC/DC_leu_ generated with Pici-_PGE1_ compared to control from leukemic PBMCs in a case of AML. 3–4 × 10^6^ PBMCs were cultured in 12-multiwell-tissue-culure-plates and diluted in 2 mL serum-free X-vivo-15-medium. For the generation of DC/DC_leu_ with Pici-_PGE1_ 500 U/mL GM-CSF and 250 U/mL IL-4 were added on day 0. After 6–7 days, 10µg/mL Picibanil a lysis product from Streptococcus pyogenes, which has unspecific immune modulatory effects and 1 µg/mL PGE_1_ were added. No response modifiers were added to the control culture. Cells were harvested after 7–10 days of incubation. Half medium exchange was carried out after 3–4 days. x-axis: CD80; y-axis: CD34.

To quantify generated DC_leu_, the cells were stained with patient-specific blast-staining antibodies (e.g., CD15, CD34, CD65, and CD117), according to diagnostic reports in combination with DC-staining antibodies (e.g., CD80, CD83, CD86, CD206, and CD209), which were not expressed on blasts before culture. For the analysis and quantification of DCs and DC_leu_ in the total- or in subtype-cell fractions after DC/DC_leu_-cultures, we used a refined gating strategy [16,17]. DC/DC_leu_ subgroup analyses were only conducted in cases with ≥10% DCs in the PBMC- and WB-fractions (premise for analysis). DC_leu_ were quantified in the total cell fraction (DC_leu_/PBMC or WB), in the DC-fraction (DC_leu_/DC^+^) or in the blast fraction, to quantify the amount of blasts converted to DC_leu_ (DC_leu_/bla^+^). The workflow and FACS analysis of DC/DC_leu_-generation with Pici-_PGE1_ as compared to control is exemplarily illustrated in Figure 5 The amount of mature DCs [DC co-expressing the migration marker CCR7 (CD197)] in the DC fraction after culture (DC_mig_/DC^+^) was quantified in the cases with ≥10% DC^+^/cells. A refined gating strategy was used to detect non-converted proliferating blasts in the PBMC- or WB-fractions (Bla_prol_/PBMC or WB) after DC/DC_leu_-culture [19]. The proliferating blasts were characterized by the co-expression of CD71 or IPO-38 without the co-expression of DC-markers. (Table 5). 

### 4.6. Mixed-Lymphocyte-Culture (MLC) of T Cell-Enriched Immunoreactive Cells with WB-Stimulator-Cell-Suspensions, Preincubated or Not Preincubated with DC/DC_leu_-Generating Protocols

1 × 10^6^ CD3^+^T cells (effector cells) from AML patients were co-cultured in 24-multiwell-tissue-culture-plates (ThermoFisher Scientific, Darmstadt, Germany) with a stimulator cell suspension containing approximately 2.5 × 10^5^ DC/DC_leu_ (MLC^WB-DC^), which were generated with different DC/DC_leu_-generating protocols from leukemic WB or PBMCs. A MLC of T cell enriched immunoreactive cells with a stimulator cell suspension without preincubation with different DC/DC_leu_-generating protocols (MLC^WB^) severed as a control. The total volume of the cell culture was adjusted to 1 mL with RPMI-1640 medium (Biochrom, Berlin, Germany) containing 1% Penicillin (Biochrom, Berlin, Germany) and 50 U/mL Interleukin 2 (IL-2, PeproTech, Berlin, Germany). After 2–3 days, 50 U/mL IL-2 was added to all cultures. PBMC-cultures contained 15% human serum (Healthcare Europa GmbH, Vienna, Austria). The cells were harvested after 6–7 days and they were used for cytotoxicity-fluorolysis-assay, as described below. Before and after culture different T cell subsets in MLC were quantified by flow cytometry (Table 5).

### 4.7. Cytotoxicity (Fluorolysis) Assay

A Fluorolysis assay was performed to analyze the blast lytic activity of T cell-enriched immunoreactive cells after MLC^WB-DC^ and MLC^WB^ [25]. Therefore, effector cells were co-cultured 1:1 with thawed blast-containing target cells for 3 and 24 h in standard laboratory conditions, as described above. As a control effector- and target-cells were cultured separately for the same time and mingled on ice shortly before flow cytometric analyses were carried out. Before culture, the target-cells were stained for 15 min. with FITC-, PE-, or APC-conjugated blast specific target cell antibodies. To evaluate viable cells and the lytic activity of effector cells, the cultures were harvested after 3 h and 24 h and then resuspended in PBS containing 7AAD (Becton Dickson, Heidelberg, Germany) and a defined number of Fluorosphere beads (Beckman Coulter, Krefeld, Germany). A refined gating was used to analyse blast lytic activity (anti-leukemic activity) [25]. Therefore, viable target cells were gated in a Forward Scatter (FSC) 7AAD^−^ gate. The cells were analyzed with a fluorescence-activated cell sorting Flow-Cytometer (FACSCalibur^TM^) and Cell-Quest-data-acquisition and analysis software (Becton Dickson, Heidelberg, Germany). The lytic activity of effector cells was calculated and defined as the difference in the percentage of viable target cells in the culture with co-cultured effector and target cells (for 3 h and 24 h) as compared to control. 

### 4.8. Enzyme-Linked Immunosorbent Assay (ELISA)

Serum and WB-DC/DC_leu_-culture supernatants were analyzed for the concentration of Interleukin 10 (IL-10), Interleukin-17A (IL-17A), and Monocyte Chemoattractant Protein-1 (MCP-1, also known as CC-chemokine ligand 2, CCL-2) while using the human IL-10 (detection limit: 1.0 pg/mL), IL-17A (detection limit: 0.5 pg/mL) and MCP-1 (detection limit: 2.3 pg/mL) immunoassay kits (DRG Instruments GmbH, Marburg, Germany). The samples were evaluated with a Tristar LB941 ELISA reader (Berthold Company, Bad Wildbach, Germany) and the concentrations of the different cytokines were evaluated with the corresponding standard curve.

### 4.9. Statistical Methods

Data were presented as mean ± standard-deviations. The cytokine-concentrations were presented as median and the corresponding range. Statistical comparisons of two groups were performed while using the two-tailed t test (in cases with data normally distributed) and the Mann-Whitney-Wilcoxon-Test (in cases with data not normally distributed). Statistical analyses were performed with Microsoft Excel 2013^®^ (Microsoft, Redmond, Washington, USA) and SSPS Statistic 24 software^©^ (IBM, Armonk, USA). The differences were considered as ‘not significant’ in cases with *p* values >0.1, as ‘borderline significant’ (significant*), with *p* values between 0.1 and 0.05, as ‘significant’ (significant**) with *p* values between 0.05 and 0.005 and as ‘highly significant’ (significant***) with *p* values <0.005. The figures were created with GraphPad Prism7^©^ (GraphPad Software, California, USA). 

## 5. Conclusion: DC/DC_leu_ Based Treatment Protocols for AML-Patients

We developed a DC/DC_leu_-generating protocol Pici-_PGE1_ and demonstrated (by replacing PGE_2_ with PGE_1_) that the efficiency of sufficient DC/DC_leu_ generation is superior from leukemic PBMCs. Furthermore, the PGE_1_-containing Kit M is a reliable tool for generating ex vivo mature DC/DC_leu_ from healthy and leukemic WB, containing the complete patient individual soluble and cellular microenvironment. These ‘new PGE_1_ generated’ DC/DC_leu_ reliably (re-) activate immunoreactive cells, improve the overall ex vivo anti-leukemic activity, and influence cytokine-release-profiles compared to the PGE_2_-containing Kit K. 

We conclude that

(1) PGE_1_-containing protocols qualify to generate and to maturate monocyte derived DCs from healthy or even patients’ PBMCs ex vivo that could, in consequence, be manipulated (e.g., pulsed with LAA) and (re-) administrated to the patients as a vaccine.
(2) PGE_1_-containing protocols qualify to produce ex vivo DC_leu_ from leukemic PBMCs. These DC/DC_leu_ could be (re-) administered to the patient in the course of an adaptive cell transfer.
(3) GM-CSF, PGE_1_, and Picibanil are drugs that are approved for human treatment, and so we conclude that, for example, the PGE_1_-containing Kit M qualify to convert (residual) myeloid blasts to DCs and DC_leu_ in vivo after the application of Kits to AML-patients. This could contribute to stabilize remission or the disease by presentation of the complete leukemic antigen repertoire to T cells and other immunoreactive cells independent of mutation or transplantation status, cytogenetic markers, FAB-classification, as well as sex or age of the patients.
(4) In vivo trials with PGE_1_-containing Kits (in animals and humans with AML) have to be performed to study safety, the efficiency of DC/DC_leu_-generation, the mediation of anti-leukemic reactions, and the establishment of immunological effects in vivo.

## 6. Patent

HMS is the inventor of the European Patent 15 801 987.7-1118 ‘Use of immunomodulatory Kits for immunotherapeutic treatment of patients with myeloid leukemia’s’. No financial conflicts of interest have to be declared.

## Figures and Tables

**Table 1:** Compositions of DC/DC_leu_-generating protocols.

DC/DC_leu_-Generating Protocols	Composition	Concentration	Sources of DC/DC_leu_	Mode of Action	Culture Time	Reference
Picibanil-_PGE1_	GM-CSF	500 U/mL	PBMC WB	**GM-CSF**: induction of myeloid (DC-) differentiation **IL-4**: induction of DC-differentiation **Picibanil (OK-432)**: lysis product from streptococcus pyogenes; stimulates DC-differentiation **PGE_2_**: increases CCR7-expression and enhances DC-migration **PGE_1_**: effects are comparable to PGE_2_	7–10 days	
(Pici-_PGE1_)	IL-4	250 U/mL				
	OK-432	10 µg/mL				
	PGE_1_	1 µg/mL				
Picibanil-_PGE2_	GM-CSF	500 U/mL	PBMC WB		7–10 days	[17,20]
(Pici-_PGE2_)	IL-4	250 U/mL				
	OK-432	10 µg/mL				
	PGE_2_	1 µg/mL				
Kit M^#^	GM-CSF	800 U/mL	WB		7–10 days	[47]
	PGE_1_	10 µg/mL				
Kit K^#^	GM-CSF	800 U/mL	WB		7–10 days	[47]
	PGE_2_	1 µg/mL				
Kit I^#^	GM-CSF	800 U/mL	WB		7–10 days	[47]
	OK-432	1 µg/mL				

DC dendritic cells; DC_leu_ dendritic cells of leukemic origin; GM-CSF granulocyte macrophage colony stimulating factor; IL-4 interleukin 4; OK-432 picibanil; PGE_2_ prostaglandin E_2_; PGE_1_ prostaglandin E_1_; PBMC peripheral blood mononuclear cells, WB whole blood; ^#^ 15 801 987.7-1118 European Patent.

**Table:** (**A**)

	MLC^WB-DC Kits^	MLC^WB^	Uncultured Cells	*p*-Values		
	% of Cells in the Corresponding Subgroup			MLC^WB-DC Kits^ vs. MLC^WB^	MLC^WB-DC Kits^ vs. Uncultured Cells	MLC^WB^ vs. Uncultured Cells
**T Cell Subtypes**						
CD3^+^CD4^+^/CD3^+^	44.7 ± 16.1	57.0 ± 18.6	**63.6 ± 21.4**	<0.08*	<0.02**	<0.44
CD3^+^CD8^+^/CD3^+^	**55.3 ± 16.1**	43.0 ± 18.6	36.4 ± 21.4	<0.08*	<0.02**	<0.44
**Proliferating T Cells**						
CD3^+^CD71^+^/CD3^+^	**22.8 ± 18.3**	17.8 ± 9.2	1.1 ± 0.6	<0.3	<0.000001***	<0.00001***
CD3^+^CD69^+^/CD3^+^	**23.0 ± 16.7**	18.4 ± 15.5	3.1 ± 4.4	<0.4	<0.00001***	<0.009***
**Non-Naïve or Naïve T cells**						
CD3^+^CD45RO^+^/CD3^+^	**73.5 ± 15.9**	67.0 ± 16.9	48.8 ± 15.6	<0.3	<0.0003***	<0.02**
CD3^+^CD8^+^CD45RO^+^/CD3^+^	**34.6 ± 13.4**	26.4 ± 14.6	16.8 ± 12.4	<0.13	<0.001***	<0.14
CD3^+^CD4^+^CD45RO^+^/CD3^+^	38.9 ± 17.3	**40.7 ± 16.1**	32.0 ± 12.5		<0.19	<0.17
CD3^+^CD45RO^−^ ^(including subsets)^	26.2 ± 15.6	32.7 ± 16.9	**51.1 ± 15.5**	<0.3	<0.0003***	<0.02**
**Effector (Memory) T Cells**						
CD3^+^CCR7^−^CD45RO^+^/CD3^+^	**62.8 ± 18.9**	55.5 ± 21.8	36.8 ± 16.6	<0.23	<0.0005***	<0.04**
CD3^+^ CCR7^+^CD45RO^+^/CD3^+^	10.3 ± 14.0	11.5 ± 15.5	11.9 ± 16.7	<0.84	<0.79	<0.95

**Table:** (**B**)

	MLC^WB-DC Kit M^	MLC^WB-DC Kit K^
**T Cell Subtypes**		
CD3^+^CD8^+^/CD3^+^	59.4 ± 13.9	59.0 ± 14.3
CD3^+^CD4^+^/CD3^+^	40.6 ± 13.9	41.0 ± 14.3
**Proliferating T Cells**		
CD3^+^CD71^+^/CD3^+^	20.7 ± 12.1	19.7 ± 13.5
**Non-Naïve or Naïve T Cells**		
CD3^+^CD45RO^+^/CD3^+^	71.8 ± 17.7	74.6 ± 16.4

MLC mixed lymphocyte culture; MLC^WB^ mixed lymphocyte culture with WB not preincubated with DC/DC_leu_-_generating_ protocols; MLC^WB-DC^ mixed lymphocyte culture with WB preincubated with DC/DC_leu_-generating protocols, highest average values are presented in bold figures.

**Table 3:** Characteristics of AML-patients and healthy volunteers included in this study are presented.

			Age at		Subtype		Blasts	Conducted	Conducted
	Stage	Pat. #	dgn.	Sex	FAB	Blast Phenotype (CD)	in PB %	DC/DC_leu_-Cultures	Experiments
**AML**	**First Diagnosis**	P1419	64	f	p/M1	**34**, **117**, 33, 15, 13	93	PBMC	
		P1426	61	f	s/M5	**34**, **117**, 64, 33, 13	40	PBMC, WB	MLC^WB^, MLC^WB-DC^, CTX
		P1430	79	m	p/M5	**34**, **117**, 33, 13	70	PBMC, WB	MLC^WB^, MLC^WB-DC^, CTX
		P1432	34	m	p/M5	**34**, 64, 33, 13	81	PBMC, WB	MLC^WB^, MLC^WB-DC^, CTX
		P1434	61	f	s/n.d.	**34**, **117**, 64, 56, 33, 13, 7	61	PBMC, WB	MLC^WB^, MLC^WB-DC^, CTX
		P1439	61	f	s/M5	**34**, **117**, 33, 13	17	PBMC, WB	MLC^WB^, MLC^WB-DC^
		P1441	60	m	s/M4	**117**, 65, 64, 33, 13, 14,	81	PBMC, WB	MLC^WB^, MLC^WB-DC^, CTX
		P1442	73	f	s/M4	**117**, 138, 61, 33	14	PBMC, WB	MLC^WB^, MLC^WB-DC^, CTX
		P1443	64	m	p/n.d.	**34**, **117**, 33, 13	50	PBMC, WB	
		P1447	21	m	p/M5	**56**, **33**, 45	65	WB	CTX
		P1449	78	m	s/n.d.	**15**, **65**, 64, 45, 4	62	WB	MLC^WB^, MLC^WB-DC^, CTX
		P1452	44	m	p/n.d.	**34**, **117**, 45, 33, 13	55	PBMC, WB	CTX
		P1453	54	f	p/M4	**15**, 64, 56, 33, 14	52	PBMC, WB	MLC^WB^, MLC^WB-DC^
		P1454	60	f	s/n.d.	**34**, **117**, 61, 20	33	WB	
		P1459	54	m	p/M4	**56**, 64, 38, 33, 11c, 11b	14	PBMC, WB	MLC^WB^, MLC^WB-DC^, CTX
		P1460	78	f	p/M4	**15**, **34**, 117	68	PBMC, WB	MLC^WB^, MLC^WB-DC^
		P1462	49	f	p/M5	**34**, 56, 64, 45, 33, 13	60	WB	
		P1466	47	f	p/n.d.	**34**, **117**, 33, 15, 13	15	PBMC	
		P1471	40	m	p/M1	**34**, **117**, 33, 13	69	PBMC, WB	
		P1472	33	f	p/M2	**117**, **34**, 15, 13	30	WB	
	**Relapse Before or after HSCT**	P1474	70	m	p/n.d.	**117**, **34**, 56, 33	80	PBMC, WB	
		P1475	77	m	s/n.d.	**117**, **34**, 33, 13	20	PBMC, WB	
		P1455	63	m	s/n.d.	**34**, **117**, 13	12	WB	
		P1463	60	f	s/n.d.	**34**, 56, 33, 13, 2	8	PBMC, WB	CTX
		P1469	49	m	p/M4	**34**, **117**, 65, 33, 13	94	PBMC	
		P1470	67	m	p/n.d.	**56**, **117**, 34, 33, 13,	9	PBMC, WB	
	**Persisting Disease**	P1464	72	m	s/n.d.	**34**, **117**, 71, 20	44	PBMC, WB	MLC^WB^, MLC^WB-DC^, CTX
		P1467	59	f	s/n.d.	**34**, **117**, 33, 15, 13	30	PBMC, WB	
		P1468	66	m	p/n.d.	**117**, 56, 34, 33	75	PBMC, WB	
**Healthy**		P1418	22	m				PBMC, WB	
		P1420	26	f				PBMC, WB	
		P1421	27	f				PBMC	
		P1422	20	f				PBMC, WB	
		P1428	56	f				PBMC, WB	
		P1429	22	f				WB	
		P1436	25	m				PBMC, WB	
		P1438	31	f				PBMC, WB	
		P1445	27	f				PBMC, WB	
		P1446	25	m				PBMC, WB	

Pat.# Patient’s number; f female; m male; p primary AML; s secondary AML; FAB French-American-British classification; PB peripheral blood; n.d. no data; dgn. diagnosis; PBMC peripheral blood mononuclear cells; WB whole blood; HSCT hematopoietic stem cell transplantation; MLC^WB^ mixed lymphocyte culture with WB not preincubated with DC/DC_leu_-generating protocols; MLC^WB-DC^ mixed lymphocyte culture with WB preincubated with DC/DC_leu_-generating protocols; CTX cytotoxicity (fluorolysis) assay; bold blasts markers used to detect DC_leu_ at the day of harvest

**Table 4:** Cellular composition of Acute myeloid leukemia (AML) and healthy samples.

	Cell Type	Average of Cells in WB/PBMCs (%)	Range of Cells in WB/PBMCS (%)
**AML**	Leukemic blasts *	46.2/45.3	8.0–81.0/8.0–93.0
	CD3+T cells	9.9/6.3	1.5–23.3/0.4–24.0
	CD19+B cells	2.8/2.4	0.1–8.4/0.2–7.6
	CD56+CD3-NK-cells	4.8/1.9	0.4–9.8/0.2–6.3
	CD14+monocytes	3.4/1.8	0.1–11.5/0.1–5.7
**Heathy**	CD14+monocytes	5.6/7.0	4.4–8.5/2.6–12.7
	CD3+T cells	19.4/36.3	13.6–26.3/21.1–46.7
	CD56+CD3-NK-cells	3.4/4.8	2.3–6.9/3.5–6.6
	CD19+B cells	2.3/6.0	0.8–4.8/1.7–11.8

* expression of CD15^+^, CD33^+^, CD34^+^, CD65^+^ and/or CD117^+^; AML acute myeloid leukemia; WB whole blood; PBMCs peripheral blood mononuclear cells.

**Table 5:** Subtypes of DC/DC_leu_ and T cells as evaluated by flow cytometry.

	Names of Subgroups	Referred to	Surface Marker	Abbreviation	Explanatory Note Premise for Analysis	Reference
**DC/DC_leu_**	leukemic blasts	cells (PBMC, WB)	CD15, CD34, CD65, CD117	Bla^+^/cells [PBMC, WB]		[16]
	dentritic cells	cells (PBMC, WB)	CD80, CD83, CD86, CD206, CD209	DC^+^/cells [PBMC, WB]		[16]
	DC_leu_ in DC fraction	DC^+^	DC^+^Bla^+^	DC_leu_/DC	≥ 10% DC^+^ in cells	[16]
	blasts converted to DC_leu_	Bla^+^	DC^+^Bla^+^	DC_leu_/Bla^+^	≥ 10% DC^+^ in cells	[16]
	leukemia derived DC	cells (PBMC, WB)	DC^+^Bla^+^	DC_leu_/cells [PBMC, WB]	≥ 10% DC^+^ in cells	[16]
	migratory mature DC in DC fraction	DC^+^	DC^+^CCR7^+^	DC_mig_/DC	≥ 10% DC^+^ in cells	[25]
	proliferating blasts	cells (PBMC, WB)	Bla^+^DC^−^CD71^+^	Bla_prol-CD71_/cells [PBMC, WB]		[19]
	proliferating blasts	cells (PBMC, WB)	Bla^+^DC^−^IPO-38^+^	Bla_prol-IPO38_/cells [PBMC, WB]		[19]
**T cell subsets**	CD3^+^ pan T cells	gated cells	CD3^+^	CD3^+^/cells		[27]
	CD4^+^ coexpressing T cells	CD3^+^	CD3^+^CD4^+^	CD4^+^CD3^+^/CD3^+^	CD4^+^ T cells	[27]
	CD8^+^ coexpressing T cells	CD3^+^	CD3^+^CD8^+^	CD8^+^CD3^+^/CD3^+^	CD8^+^ T cells	[27]
	early proliferating T cells	CD3^+^	CD3^+^CD69^+^	T_prol_/CD3^+^	proliferating T cells	[27]
	proliferating T cells	CD3^+^	CD3^+^CD71^+^	T_prol_/CD3^+^	proliferating T cells	[27]
	naive T cells	CD3^+^	CD3^+^CD45RO^−^	T_naive_/CD3^+^	Unprimed T cells	[28]
	naive CD4^+^ T cells	CD3^+^	CD3^+^CD4^+^CD45RO^−^	T_CD4-naive_/CD3^+^	Unprimed CD4^+^ T cells	[28]
	naive CD8^+^ T cells	CD3^+^	CD3^+^CD8^+^CD45RO^−^	T_CD8-naive_/CD3^+^	Unprimed CD8^+^ T cells	[28]
	non-naive T cells	CD3^+^	CD3^+^CD45RO^+^	T_non-naive_/CD3^+^	Memory + effector T cells	[28]
	non-naive CD4^+^ T cells	CD3^+^	CD3^+^CD4^+^CD45RO^+^	T_CD4-non-naive_/CD3^+^	Memory + effector CD4^+^ T cells	[28]
	non-naive CD8^+^ T cells	CD3^+^	CD3^+^CD8^+^CD45RO^+^	T_CD8-non-naive_/CD3^+^	Memory + effector CD8^+^ T cells	[28]
	central (memory) T cells	CD3^+^	CD3^+^CCR7^+^CD45RO^+^	T_cm_/CD3^+^	Long-term immunity	[28]
	effector (memory) T cells	CD3^+^	CD3^+^CCR7^−^CD45RO^+^	T_eff_T_ems_/CD3^+^		[28]

Surface marker combinations for the analysis of DC and DC_leu_ (including subsets) and T cell subtypes after flow cytometric staining with fluorochrome-labelled-antibodies are given. Cells were analyzed before and after different cultures.
